# *Fasciola gigantica* tegumental calcium-binding EF-hand protein 4 exerts immunomodulatory effects on goat monocytes

**DOI:** 10.1186/s13071-021-04784-5

**Published:** 2021-05-22

**Authors:** Muhammad Ehsan, Rui-Si Hu, Jun-Ling Hou, Hany M. Elsheikha, Xiao-Dong Li, Pan-Hong Liang, Xing-Quan Zhu

**Affiliations:** 1 State Key Laboratory of Veterinary Etiological Biology, Key Laboratory of Veterinary Parasitology of Gansu Province, Lanzhou Veterinary Research Institute, Chinese Academy of Agricultural Sciences, Lanzhou, 730046 Gansu China; 2 Department of Parasitology, Faculty of Veterinary and Animal Sciences, The Islamia University of Bahawalpur, Bahawalpur, 63100 Punjab Pakistan; 3 Faculty of Medicine and Health Sciences, School of Veterinary Medicine and Science, University of Nottingham, Sutton Bonington Campus, Loughborough, LE12 5RD UK; 4 College of Veterinary Medicine, Shanxi Agricultural University, Taigu, 030801 Shanxi China; 5 Key Laboratory of Veterinary Public Health of Higher Education of Yunnan Province, College of Veterinary Medicine, Yunnan Agricultural University, Kunming, 650201 Yunnan China

**Keywords:** *Fasciola gigantica*, Tegumental calcium-binding EF-hand protein 4, Immune responses, Monocytes, Host-parasite interactions

## Abstract

**Background:**

The liver fluke *Fasciola gigantica* secretes excretory-secretory proteins during infection to mediate its interaction with the host. In this study, we investigated the immunomodulatory effects of a recombinant tegumental calcium-binding EF-hand protein 4 of *F. gigantica* (rFg-CaBP4) on goat monocytes.

**Methods:**

The rFg-CaBP4 protein was induced and purified by affinity chromatography. The immunogenic reaction of rFg-CaBP4 against specific antibodies was detected through western blot analysis. The binding of rFg-CaBP4 on surface of goat monocytes was visualized by immunofluorescence assay. The localization of CaBP4 within adult fluke structure was detected by immunohistochemical analysis. The cytokine transcription levels in response to rFg-CaBP4 were examined using ABI 7500 real-time PCR system. The expression of the major histocompatibility complex (MHC) class-II molecule (MHC-II) in response to rFg-CaBP4 protein was analyzed using Flow cytometry.

**Results:**

The isopropyl-ß-D-thiogalactopyranoside-induced rFg-CaBP4 protein reacted with rat sera containing anti-rFg-CaBP4 polyclonal antibodies in a western blot analysis. The adhesion of rFg-CaBP4 to monocytes was visualized by immunofluorescence and laser scanning confocal microscopy. Immunohistochemical analysis localized native CaBP4 to the oral sucker, pharynx, genital pore, acetabulum and tegument of adult *F. gigantica*. Co-incubation of rFg-CaBP4 with concanavalin A-stimulated monocytes increased the transcription levels of interleukin (IL)-2, IL-4, interferon gamma and transforming growth factor-β. However, a reduction in the expression of IL-10 and no change in the expression of tumor necrosis factor-α were detected. Additionally, rFg-CaBP4-treated monocytes exhibited a marked increase in the expression of the major histocompatibility complex (MHC) class-II molecule (MHC-II) and a decrease in MHC-I expression, in a dose-dependent manner.

**Conclusions:**

These findings provide additional evidence that calcium-binding EF-hand proteins play roles in host-parasite interaction. Further characterization of the immunomodulatory role of rFg-CaBP4 should expand our understanding of the strategies used by *F. gigantica* to evade the host immune responses.

**Graphical abstract:**

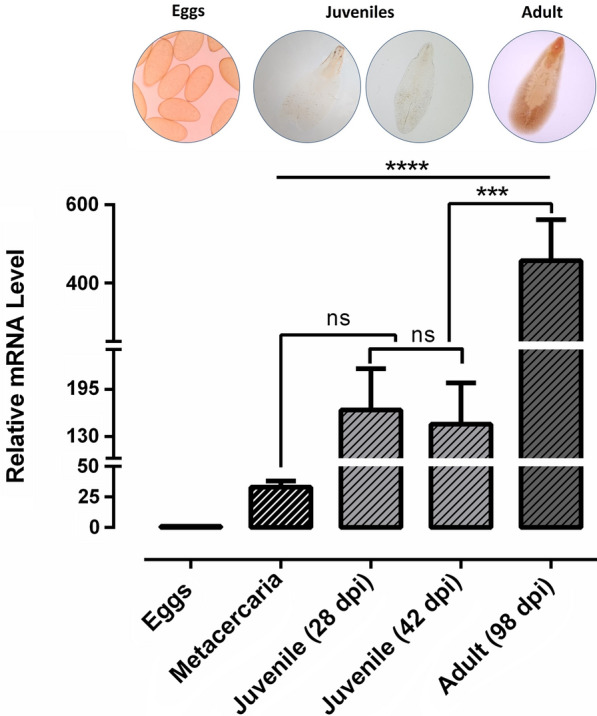

**Supplementary Information:**

The online version contains supplementary material available at 10.1186/s13071-021-04784-5.

## Background

*Fasciola hepatica* and *Fasciola gigantica* are causative agents of a trematode infection (fascioliasis) and are prevalent in temperate and tropical regions of the world, respectively [1, 2]. These liver flukes cause significant damage to the host’s tissues [3, 4]. The annual global economic losses due to fascioliasis are estimated to be US $3 billion [2]. Fascioliasis has been listed as a neglected tropical disease by the World Health Organization and is considered to be a re-emerging zoonotic disease, infecting ~ 2.4 million people and posing a risk to ~ 180 million [5]. Current strategies to control fascioliasis rely on the use of the flukicide triclabendazole; however, the emergence of resistance has become a serious problem [6]. For this reason, alternative or complementary approaches are required to enhance the control and treatment of fasciolosis.

Tegumental proteins (TegPs) belong to a family of calcium-binding proteins (CaBPs), which contain two EF-hand motifs at the N-terminal domain and a C-terminal dynein light chain-like domain [7]. CaBPs have been characterized in various helminth parasites including *F. hepatica* [8], *F. gigantica* [9], *Schistosoma mansoni* [10], *Clonorchis sinensis* [11], *Echinococcus multilocularis* [12] and *Opisthorchis viverrini* [13]. CaBPs have been categorized into 4 isoforms in *F. hepatica* and *F. gigantica* (CaBP-1–4) [14], and around 13 isoforms in *S. mansoni* [10]. The biochemical properties of CaBPs from different parasites have been investigated, and despite the considerable resemblance between structural models of all family members, differences exist in the dimerization, ion-binding and drug-binding characteristics of these apparently similar proteins [14]. This may be an indication of their pivotal role in immune mechanisms during host-parasite interactions.

CaBPs are enriched on the tegumental surface of parasites, as well as in the parenchyma and gut epithelium, and are involved in ion uptake and immune evasion via the production of immune modulatory molecules [15]. TegPs of *F. hepatica* have immune modulatory roles during fascioliasis via altering the functions of mast cells and dendritic cells (DCs), ultimately inhibiting the helper T cell 1 (Th1) immune response [16]. The tegumental surface of liver flukes plays critical functions, such as structural support, a sensory response, osmoregulation, nutrient uptake, excretion, immune modulation and evasion, and signal transduction [17]. Various parasite species, including *F. gigantica*, secrete CaBPs which induce an immunoglobulin E (IgE) immune response in the hosts [10, 15, 18]. The immunogenic potential of these proteins has been evaluated and preliminary immunization studies with some TegPs have shown that they provide significant protection against infection [10]. The CaBP family is unique to worms and thus absent in the mammalian host [14]; therefore, these proteins may provide specific targets for the development of antiparasitic therapies. Previous studies have shown that *F. hepatica* TegPs impair DC maturation and function by downregulating the production of interleukin (IL)-6, IL-10, IL-12 and tumor necrosis factor-α (TNF-α) together with CD40, CD80, CD86 and cell surface markers on DCs [19, 20]. However, the mechanisms by which these proteins interact with host cells to manipulate host immune responses remain poorly understood.

Liver fluke-secreted proteins interact with host immune cells, such as goat peripheral blood mononuclear cells (PBMCs), to manipulate their immune functions [21]. Tegumental calcium-binding EF-hand protein 4 (CaBP4) is an important constituent of *F. gigantica* excretory-secretory proteins and may have immunoregulatory roles during *F. gigantica* infection [22]. In the present study, we cloned and characterized *F. gigantica* CaBP4 (Fg-CaBP4) and examined its immunoregulatory effects on goat monocytes in vitro. Our findings showed that Fg-CaBP4 influenced the expression of cytokines and immune factors in goat monocytes and confirmed the immunogenic potential of Fg-CaBP4.

## Methods

### Animals used in the experiments

A local crossbred goat (1-year-old) was housed in strict hygienic conditions at the Laboratory Animal Center of Lanzhou Veterinary Research Institute, Chinese Academy of Agriculture Sciences. To ensure that the goat had no parasitic infection prior to inclusion in the experiment, a fecal examination was carried out daily for a short period and the animal was given albendazole (10 mg/kg body weight) twice at a fortnightly interval. The goat was confirmed to be free of helminth infections. For the production of antibodies, four female Sprague–Dawley rats (~ 150 g) were purchased from the Laboratory Animal Center of Lanzhou Veterinary Research Institute and raised under pathogen-free conditions with a sufficient supply of food and water. For the immunological studies, three individual experiments—each consisting of three replicates—were carried out by isolating monocytes from the same goat.

### Cell isolation and culture

Peripheral blood samples were collected from the jugular vein of the goat into a Vacutainer and brought to the laboratory for cell isolation as previously described [23]. The blood sample was mixed with an equal volume (1:1) of Ca^2+^/Mg^2+^-free phosphate buffer saline (PBS; pH 7.4) and PBMC isolation was carried out using a standard Ficoll-Hypaque gradient centrifugation method (GE Healthcare, Munich, USA) [24] and a goat PBMC isolation kit (TBD, Tianjin, China). The PBMCs were carefully transferred to new tubes and washed three times with PBS. To isolate monocytes, PBMCs were seeded in 6-well flat-bottomed plastic tissue culture plates (Costar, Cambridge, MA) containing Roswell Park Memorial Institute 1640 (RPMI-1640) culture medium (Gibco Life Technologies, USA) supplemented with 10% heat-inactivated fetal calf serum (fetal bovine serum; FBS), 100 mg/ml streptomycin and 100 U/ml penicillin (Gibco Life Technologies), then incubated in a humidified incubator in 5% CO_2_ at 37 °C. The monocytes that adhered to the bottom of the cell culture plate were collected [25] and their number was adjusted to achieve the required cell density. Non-adherent cells were removed by multiple steps of washing with PBS. For all experiments, the viability of monocytes was > 95% as determined by a trypan blue exclusion assay.

### Source of the parasite

The collection of *F. gigantica* flukes was carried out as previously described [26]. Briefly, 8- to 10-month-old buffaloes were kept indoors in clean sheds with sufficient provision of water and rice plants. To ensure that the buffaloes were free of any previous helminth infection, they were treated orally with triclabendazole 5% weight/volume (w/v) suspension. The animals were infected with viable metacercariae and their health was monitored twice daily throughout the entire infection period. Ninety-eight days after infection the buffaloes were slaughtered and the parasites were collected from the gall bladder and stored at − 80 °C until analysis. The eggs were harvested by elution from adult flukes collected from buffalo liver as described previously [27]. For collection of *F. gigantica* juveniles, mice were infected by oral gavage with 30 viable metacercariae in 1 ml of PBS and maintained in a containment laboratory, with room temperature (22 ± 0.5 °C) under a 12/12-h light/dark cycle. All mice were fed a commercial diet with access to purified water ad libitum. Following infection, mice were monitored for the development of clinical signs, such as ruffled fur and physical inactivity. At 28 and 42 days post-infection (dpi), mice were anesthetized by intraperitoneal injection with 100 μl xylazine (20 mg/ml) and ketamine (1 mg/ml) in PBS, and liver tissues were harvested and washed in PBS to collect *F. gigantica* juveniles. In this study, buffaloes were infected with viable metacercariae to obtain adult *F. gigantica* for RNA isolation, protein localization and to determine messenger RNA (mRNA) transcription levels of CaBP4. For the immunological studies, PBMCs were isolated from the infection-free goat. Goat PBMCs and monocytes were used in the present and previous studies [28, 29] as models to investigate the influence of *F. gigantica*-derived proteins on the immune-associated cellular functions of innate immune cells.

### RNA isolation and first-strand complementary DNA synthesis

The *F. gigantica* adult flukes were collected, as described above, and RNA isolation was carried out using the Trizol method (Invitrogen, Shanghai, China) according to the manufacturer’s directions. The flukes were chopped and homogenized for 30 min in a pre-chilled pestle and mortar containing 1 ml Trizol; 200 μl of trichloromethane was then added and the mixture was centrifuged at 10,000×*g* for 10 min at 4 °C. The RNA in the supernatant was precipitated by adding 250 μl of isopropyl alcohol (0.25 volume/1 ml Trizol) and incubating at − 20 °C for 30 min, followed by centrifugation at 10,000×*g* at 4 °C for 10 min. Finally, the RNA pellet was dried, washed with 70% ethanol and re-suspended in diethyl pyrocarbonate-treated water. The complementary DNA (cDNA) was reverse transcribed using a cDNA Synthesis Kit (Takara Biotechnology, Dalian, China) according to the manufacturer’s instructions and maintained at − 20 °C for further use.

### Cloning of the* Fg*-*CaBP4* gene

The following primers were designed using Primer Premier 5.0v software based on the complete open reading frame (ORF) of Fg-CaBP4 (GenBank: JN604670.1): Fg-CaBP4 forward 5ʹ-GGATCCATGGGCGAAGTGGCACTAG-3ʹ and Fg-CaBP4 reverse 5ʹ GAGCTCCTAATTGCTCGGTGTGCG-3ʹ (restriction sites, BamHI and SacI, are underlined). The target region including the restriction sites, was amplified using reverse transcription-polymerase chain reaction (RT-PCR). The amplified product was electrophoresed and purified using a gel extraction kit (Omega Bio-Tek, GA) according to the manufacturer’s instructions. The purified fragment was then inserted into a pMD-19T cloning vector (Takara Biotechnology). The resultant constructs were transformed into *Escherichia coli* Trans5α competent cells (TransGen Biotech, Beijing) and cultured in Luria Bertini agar medium containing kanamycin antibiotic (100 μg/ml). Random clones were then collected and positive clones were confirmed by PCR followed by digestion using the BamHI and SacI restriction enzymes. Sequencing of the recombinant plasmid (pMD-19T/Fg-CaBP4) was performed by Invitrogen Biotech (Shanghai, China) and the obtained sequences were confirmed by sequence alignment using the online Blast program (https://blast.ncbi.nlm.nih.gov/Blast.cgi). The recombinant plasmid (pMD-19T/Fg-CaBP4) was digested with a pair of restriction endonuclease enzymes, BamHI and SacI. The eluted target fragment was then ligated into the expression vector pET28a(+) (Novagen, Shanghai, China) and transferred to *E. coli* (BL21) DE3 strain. The positive clones were confirmed, again using dual digestion enzymes and electrophoresis, and the pET28a-Fg-CaBP4 product was further verified by sequencing (GenScript, Shanghai, China) to ensure the accurate insertion of the *CaBP4* gene into the expression plasmid cassette.

### Bioinformatics analysis

The sequence data received from GenScript were matched to known CaBP4 sequences from other parasites available from the National Center for Biotechnology Information (NCBI) using BLASTx and BLASTp (https://blast.ncbi.nlm.nih.gov/Blast.cgi). Structural prediction of the CaBP4 protein was evaluated using the Protein Homolog/analogY Recognition Engine v2.0 (Phyre2) (http://www.sbg.bio.ic.ac.uk/~phyre2/html/page.cgi?id=index). A phylogenetic tree, based on a neighbor-joining method, was constructed and visualized using Molecular Evolutionary Genetics Analysis 6.0 software [30]. Predicting the structural characteristics of the amino acid sequence was achieved using various bioinformatics approaches, such as N-terminal signal peptides analysis (http://www.cbs.dtu.dk/services/SignalP/output.html), N-glycosylation sites prediction (http://www.cbs.dtu.dk/services/NetNGlyc/), transmembrane domain predictions (http://www.cbs.dtu.dk/services/TMHMM/), GPI modification site prediction (http://mendel.imp.ac.at/sat/gpi/gpi_server.html), T cell motifs [DNASTAR (EditSeq, Protean)], and B cell epitope prediction (http://tools.immuneepitope.org/bcell/).

### Expression and purification of recombinant Fg-CaBP4 protein

Positive clones were re-cultured in Luria Bertini medium containing a specific antibiotic at 37 °C for almost 2 h to obtain an optimal density of 0.6. Protein expression was then induced by adding 1 mM isopropyl-ß-D-thiogalactopyranoside (IPTG; Sigma-Aldrich, Shanghai, China). The culture was grown for a further 6 h and samples were collected before and at 2-h intervals following IPTG induction. The bacterial pellet was collected and sonicated. The expressed protein was run on 12% (w/v) sodium dodecyl sulfate–polyacrylamide gel electrophoresis (SDS-PAGE) and visualized with Coomassie Brilliant Blue (G-250) staining. The recombinant protein was purified by a Ni^2+^-nitrilotriacetic acid column (GE Healthcare, USA) as described previously [31]. The recombinant protein was quantified using the Bradford method [32], with bovine serum albumin as a standard, and then stored at − 20 °C for further analysis as described below.

### Production of antibodies and western blotting analysis

About 0.3 mg of purified recombinant Fg-CaBP4 (rFg-CaBP4) protein was emulsified with an equal amount of Freund's complete (1:1) solution and subcutaneously injected into Sprague–Dawley rats to generate polyclonal antibodies against rFg-CaBP4. After 2 weeks, a booster dose was given with a combination of Freund's incomplete and rCaBP4 in the same ratio and via the same dose route used in the initial injection. Two additional booster doses were given at 1-week intervals. Finally, the rats were anesthetized and blood samples were collected directly from heart punctures. The sera containing antibodies from immunized and normal rats were separated and stored at − 20 °C.

The purified rFg-CaBP4 protein (20 µg) was electrophoresed in 12% SDS-PAGE and transferred to a polyvinylidene difluoride membrane (Immobilon-P; Millipore, Billerica, MA) using an eBlot protein transfer device (GenScript, Nanjing, Jiangsu, China). Polyvinylidene difluoride membranes were treated with 5% skimmed milk (w/v) dissolved in Tris-buffered saline containing 0.05% Tween-20 at 37 °C for 1 h to block non-specific binding. The strips were washed three times with Tris-buffered saline containing 0.05% Tween-20 and incubated with rat anti-rFg-CaBP4 sera, or normal rat sera, as the primary antibody (1:300 dilutions) followed by goat anti-rat horseradish peroxidase-labeled immunoglobulin G (Santa Cruz Biotechnology, CA) as the secondary antibody (1:500 dilutions) at 37 °C for 2 h. Finally, the strips were washed and immunoreactivity was visualized within 3–5 min using 3,3′-diaminobenzidine (Sigma-Aldrich) in the dark.

### Transcriptional analysis of the* CaBP4* gene in *F. gigantica* developmental stages

In order to examine dynamic changes in the transcriptional level of *CaBP4* at different stages of the *F. gigantica* life cycle, total RNA was extracted from eggs, juveniles (28 and 42 dpi) and adults (98 dpi). The corresponding cDNAs were synthesized in accordance with the assay manufacturer’s instructions (Takara Biotechnology). Transcriptional analysis of the candidate gene was performed using the standard procedure for an ABI 7500 Real-Time PCR System (Applied Biosystems) and specific primers for the endogenous reference gene *β-actin* and target gene* Fg*-*CaBP4* (Additional file 1: Table S1). The relative transcription levels of* Fg*-*CaBP4* were calculated using the 2^−ΔΔCt^ method [33]. This experiment was performed in triplicate.

### Localization of CaBP4 protein in *F. gigantica* adults

Adult *F. gigantica* flukes collected at 98 dpi were fixed in 4% formaldehyde solution for 24 h then transferred to a solution of 30% sucrose in PBS for 2 days. The flukes were then dried, dipped in Tissue-Tek O.C.T. Compound (Sakura Finetek, Torrance, CA) and snap frozen in liquid nitrogen. Sectioning of the parasites was performed using a Cryostat CM1520 (Leica Biosystems, Nussloch, Germany), with 15-μm section thickness; the sections were preserved at − 80 °C. The slides were thawed at room temperature for 30 min and fixed in 4% formaldehyde-0.2% glutaraldehyde in PBS for 30 min. Non-specific binding sites were blocked with 10% normal goat serum in PBS for 1 h. The sections were incubated with specific rat-anti-rFg-CaBP4 antiserum (1:100 dilutions), or normal rat serum (control), overnight at 4 °C, followed by the secondary antibody (goat anti-rat IgG) coupled with cyanine dye 3 (Cy3) at 37 °C for 4 h. Nuclei were counterstained with 1.5 μM 2-(4-amidinophenyl)-6-indolecarbamidine dihydrochloride (DAPI; Sigma, St. Louis, MI) for 10 min. Finally, the slides were examined under a Leica TCS CP8 confocal microscope (Leica Microsystems, Germany).

### rFg-CaBP4 protein binding to the surface of goat monocytes

The monocytes were separated from freshly collected PBMCs, and washed with PBS (Ca^2+^/Mg^2+^-free, pH 7.4). Cells numbers were adjusted to 1 × 10^5^ cells/ml in RPMI-1640 supplemented with 10% FBS, 100 U/ml penicillin and 100 mg/ml streptomycin (Gibco Life Technologies); then incubated with rFg-CaBP4, or control buffer, at 37 °C in 5% CO_2_ for 2 h. Protein binding was examined using an immunofluorescence assay [34]. The monocytes were washed and added to poly-L-lysine-treated slides, fixed with 4% paraformaldehyde for 30 min and permeabilized with 1% Triton X-100/PBS. The monocytes were incubated with primary rat anti-rFg-CaBP4 IgG antibody (1:200 dilutions), or normal rat serum, overnight at 4 °C, followed by incubation in the secondary goat anti-rat IgG antibody (Beyotime, China) coupled with Cy3 (1:1000 dilutions) for 4 h at 37 °C. Subsequently, nuclei of monocytes were stained with DAPI (Sigma, USA), and protected from the light for 10 min. Anti-fade Fluoromount solution (Beyotime, China) was added to the slides before examination under a Leica TCS CP8 confocal microscope at 100× magnification (Leica Microsystems).

### Expression of cytokine transcripts in response to rFg-CaBP4

The monocytes were stimulated with concanavalin A (ConA; 10 μg/ml) and incubated with various doses of rFg-CaBP4 (5 μg/ml, 10 μg/ml, 20 μg/ml and 40 μg/ml) in RPMI 1640 culture medium (containing 100 U/mL penicillin, 100 μg/ml streptomycin, 2 mmol/l l-glutamine and 10% FBS) at 37 °C in 5% CO_2_ for 72 h. The cells were harvested and a PrimeScript RT reagent kit (Takara Biotechnology, CA) was used to extract RNA from cell pellets. This was followed by cDNA synthesis using ThermoScript RT and Oligo (dT) 20 primers (Invitrogen) in line with the assay manufacturer’s instructions. Transcription levels of the cytokines IL-2, IL-4, IL-10, interferon-γ (IFN-γ), transforming growth factor (TGF)-β (TGF-β1), TNF-α and endogenous reference gene (glyceraldehyde-3-phosphate dehydrogenase) were examined using an ABI 7500 Real-Time PCR System (Applied Biosystems) with the following cycling conditions: initial denaturation at 95 °C for 30 s; amplification at 95 °C for 5 s and 60 °C for 1 min and a melting curve stage at 60–95 °C. The specific primers for each cytokine gene, including the reference gene, and their amplification efficiencies are summarized in Additional file 1: Table S2. The cycle threshold (Ct) values obtained from ABI Prism 7500 software version 2.0.6 (Applied Biosystems) were used to obtain the relative gene expression values using the 2^−ΔΔCt^ method. The data were obtained from triplicate experiments.

### Expression of major histocompatibility complex molecules on goat monocytes

Major histocompatibility complex (MHC) molecule expression analysis was performed as previously described [35]. Freshly isolated monocytes (1 × 10^5^ cells/ml per well) were seeded in 24-well cell culture plates containing complete RPMI 1640 medium. The cells were incubated in different concentrations of rFg-CaBP4 (5 μg/ml, 10 μg/ml, 20 μg/ml and 40 μg/ml) or an equal volume of control buffer for 24 h at 37 °C. Subsequently, monocytes were marked with MHC class-I (MHC-I; MCA2189A647) and MHC-II (MCA2226F) monoclonal antibodies (AbD Serotec; BioRad Laboratories, CA). The results were expressed as the percentage of mean fluorescence intensity and analyzed using a FACSCalibur Flow Cytometer (BD Biosciences).

### Statistical analysis

The results are presented as means ± SD from three independent experiments. The data were analyzed using GraphPad Prism 6.0 for Windows (San Diego, CA). Multiple groups were compared and analyzed using one-way ANOVA followed by Tukey’s test. Two groups were compared using Student’s *t*-test. A value of *P* < 0.05 was taken to be statistically significant.

## Results

### Fg-CaBP4 cDNA and polypeptide

**Fig. 1a–c:**
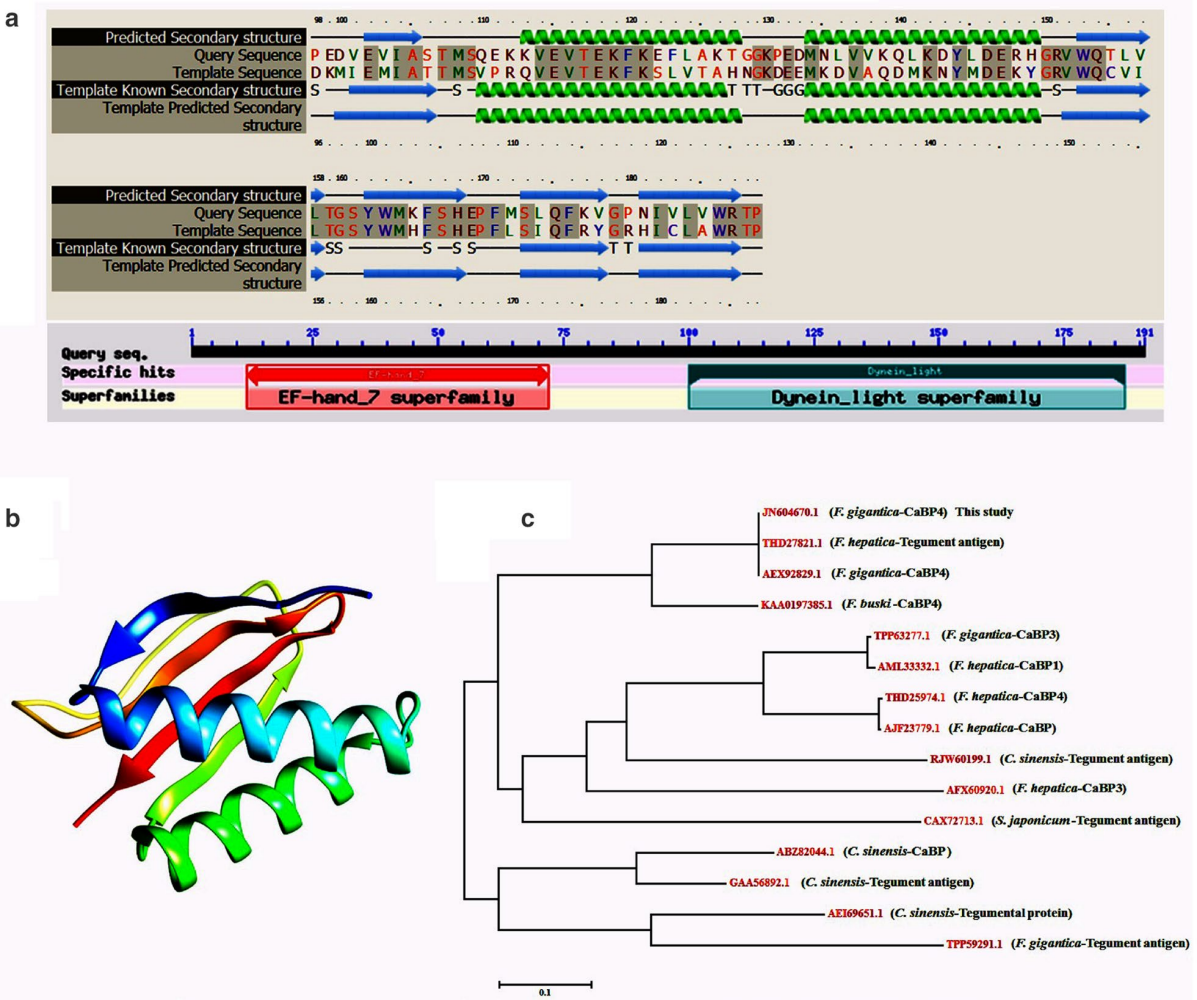
*Fasciola gigantica* tegumental calcium-binding EF-hand protein (*CaBP*) 4 (Fg-CaBP4) sequence and phylogenetic analysis. **a** The predicted protein homology and secondary structures with the best template found in the Phyre2 search tool, and BLAST search against the National Center for Biotechnology Information (NCBI) non-redundant protein sequence highlighting the two major domains, EF-hand and dynein-light chain, in the Fg-CaBP4 protein. **b** The predicted 3-dimensional model of Fg-CaBP4 shows 55% similarity to *Fasciola hepatica* CaBP2 protein (Protein Data Bank: p6422). **c** A neighbor joining phylogenetic tree showing genetic relatedness between Fg-CaBP4 and CaBPs from other helminth parasites in the NCBI database

A cDNA region corresponding to an ORF of Fg-CaBP4 polypeptide was obtained by RT-PCR and appeared as an amplicon of 576 bp in ethidium bromide-stained agarose gel. The eluted fragment was further cloned into a pMD-19T vector, followed by expression in a pET28a(+) plasmid, and confirmed by BamHI and SacI restriction site digestion. The sequencing of the target fragment indicated its accurate insertion into the expression vector, resulting in the recombinant plasmid pET28a(+)-Fg-CaBP4. The Fg-CaBP4 cloned sequence translated into 191 amino acid residues with a predicted protein size of 22,250 Da and 4.977 pI. The Phyre2 search engine predicted the presence of a CaBP domain comprising 98–189 residues (Fig. 1a) and the best template found to generate the 3D structure was *F. hepatica* CaBP2 with 55% sequence similarity (Protein Data Bank: p6422) (Fig. 1b). The amino acid sequence of Fg-CaBP4 showed high sequence similarity to other CaBPs and tegumental protein sequences from various helminth parasites available in the NCBI database. The most related identity on a BLASTp search had 96.86% similarity to Fg-CaBP4 (GenBank: AEX92829.1), 88.94% with *Fasciola hepatica* tegument-associated antigen (GenBank: THD27821.1) and 78.72% with *Fasciolopsis buski* CaBP4 (GenBank: KAA0197385.1) (Table 1). A neighbor-joining phylogenetic tree showed that Fg-CaBP4 was clustered with highly related sequences from *F. gigantica*, *F. hepatica* and other helminth species (Fig. 1c). Sequence analysis also predicted T cell motifs and B cell epitopes in the deduced protein structure. Proteins without signal peptides are unlikely to be exposed to *N*-glycosylation and thus may not be glycosylated. In addition, no signal peptide, GPI modification site or transmembrane domain was predicted in the Fg-CaBP4 protein sequence (Additional file 2: Figures S1–S5).

**Table 1:** Amino acid similarity of *Fasciola gigantica* tegumental calcium-binding EF-hand protein (*CaBP*) 4 sequence obtained in the present study to sequences of related trematode species available in the National Center for Biotechnology Information database

Description	*E*-value	Identity %	GenBank accession no.
*Fasciola gigantica* CaBP4	3e−134	96.86	AEX92829.1
*Fasciola hepatica* tegument-associated antigen	2e−129	88.94	THD27821.1
*Fasciolopsis buski* CaBP4	1e−109	78.72	KAA0197385.1
*Clonorchis sinensis* tegument antigen	7e−69	56.84	GAA56892.1
*C. sinensis* CaBP	8e−67	57.14	ABZ82044.1
*F. hepatica* calcium-binding protein	4e−63	51.40	AJF23779.1
*F. hepatica* CaBP4	6e−63	51.40	THD25974.1
*F. gigantica* CaBP3	5e−60	48.39	TPP63277.1
*F. hepatica* CaBP1	2e−59	47.85	AML33332.1
*Schistosoma japonicum* tegument antigen	1e−57	48.37	CAX72713.1
*C. sinensis* 21.6-kDa tegumental protein	3e−55	45.55	AEI69651.1
*F. gigantica* tegument antigen	4e−47	40.98	TPP59291
*C. sinensis* 21.6-kDa tegumental protein	3e−55	45.55	AEI69651.1
*F. hepatica* CaBP3	1e−49	46.15	AFX60920
*C. sinensis* tegument antigen	3e−54	44.69	RJW60199.1

### Transcriptional profiles of* CaBP4* in life cycle stages of *F. gigantica*

**Fig. 2:**
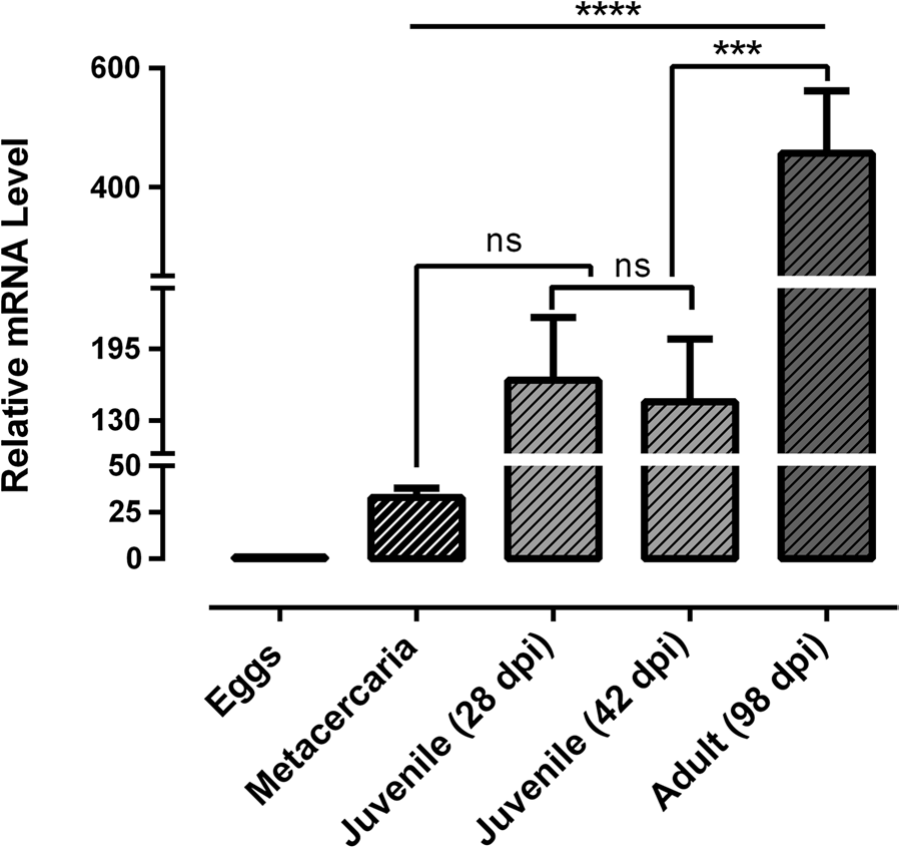
The relative expression of *CaBP4* in *F. gigantica* life cycle stages. The messenger RNA (*mRNA*) transcriptional patterns of *CaBP4* in eggs, juvenile [28 and 42 days post-infection (*dpi*)] and adult (98 dpi) flukes were examined by real-time polymerase chain reaction. The transcription value of *CaBP4* in the eggs was normalized to 1.0. The relative changes in the gene transcription ratio were normalized to the transcription of a reference gene and calculated by the 2^−ΔΔCt^ method. The data are presented as mean ± SD from three independent experiments. Data were analyzed by one-way ANOVA.* ns* Non-significant; ***P < 0.001, ****P < 0.0001

Real-time PCR analysis showed that mRNA transcription of *CaBP4* was significantly altered at different life cycle stages of *F. gigantica*. The transcription levels of *Fg-CaBP4* were observed to increase from juvenile (28 dpi and 42 dpi) to adult fluke (98 dpi) when compared with that of metacercaria. The relative quantities in juvenile (28 dpi), juvenile (42 dpi) and adults (98 dpi) were 166.6, 147.1 and 456.6, respectively; whereas the relative quantity in metacercaria was 32.97 (Fig. 2). The highest level of *Fg-CaBP4* transcription was found in adult *F. gigantica* [ANOVA, *F*_(4,10)_ = 27.60, *P* < 0.0001], when compared with that of metacercaria (Fig. 2).

### Distribution of CaBP4 in adult *F. gigantica*

**Fig. 3a, b:**
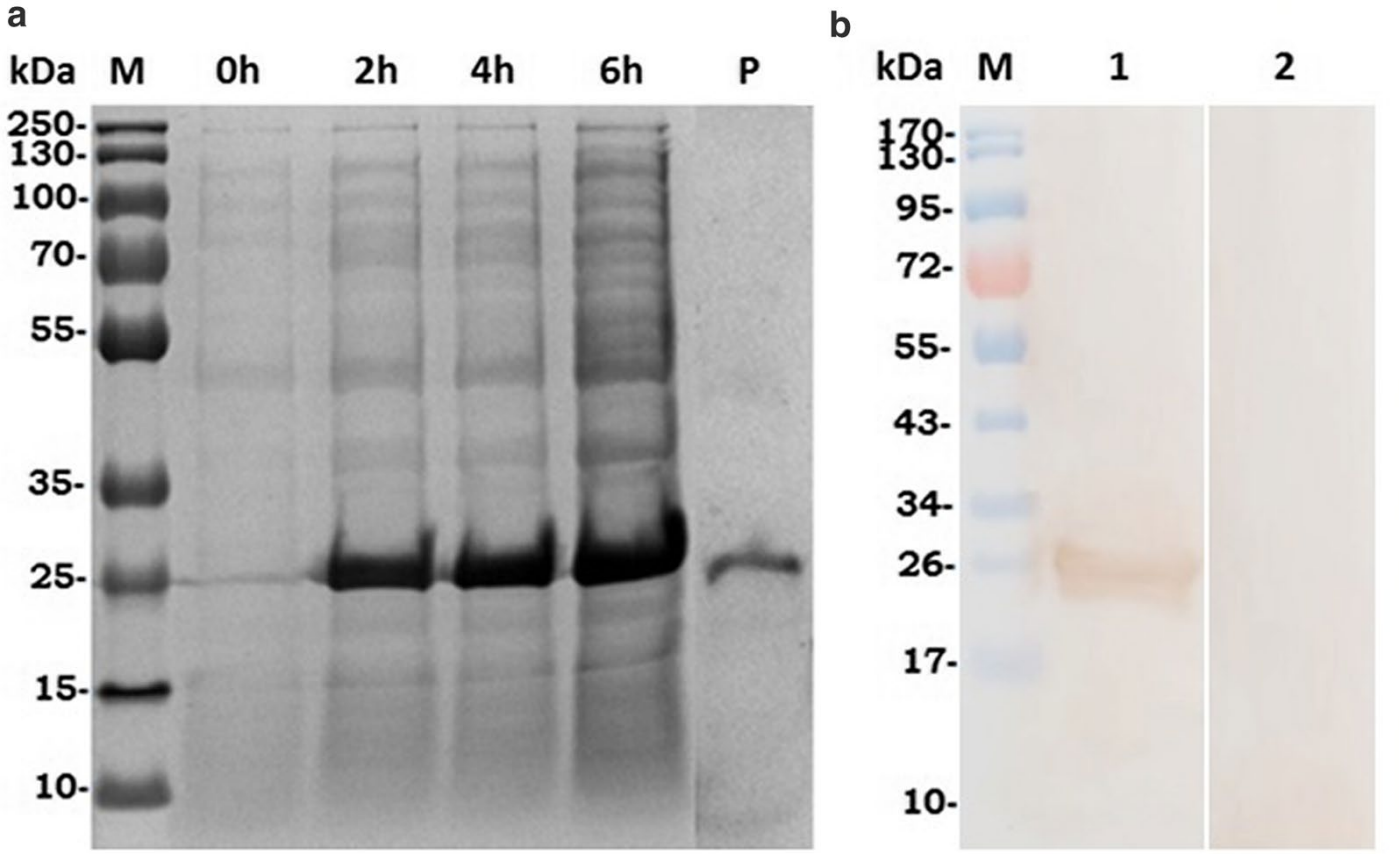
Western blotting was performed to confirm the expression of recombinant Fg-CaBP4 (rFg-CaBP4) protein [molecular weight protein ladder (*M*)]. **a** The recombinant protein expression before isopropyl-ß-D-thiogalactopyranoside induction (0 h), protein expression at different times (2, 4, 6 h) and the purified protein resolved by sodium dodecyl sulfate–polyacrylamide gel electrophoresis (*P*). **b** The purified protein was transferred to a polyvinylidene difluoride membrane which was probed with rat sera containing anti-rFg-CaBP4 antibodies (*1*) or incubated with normal rat sera (*2*)

**Fig. 4:**
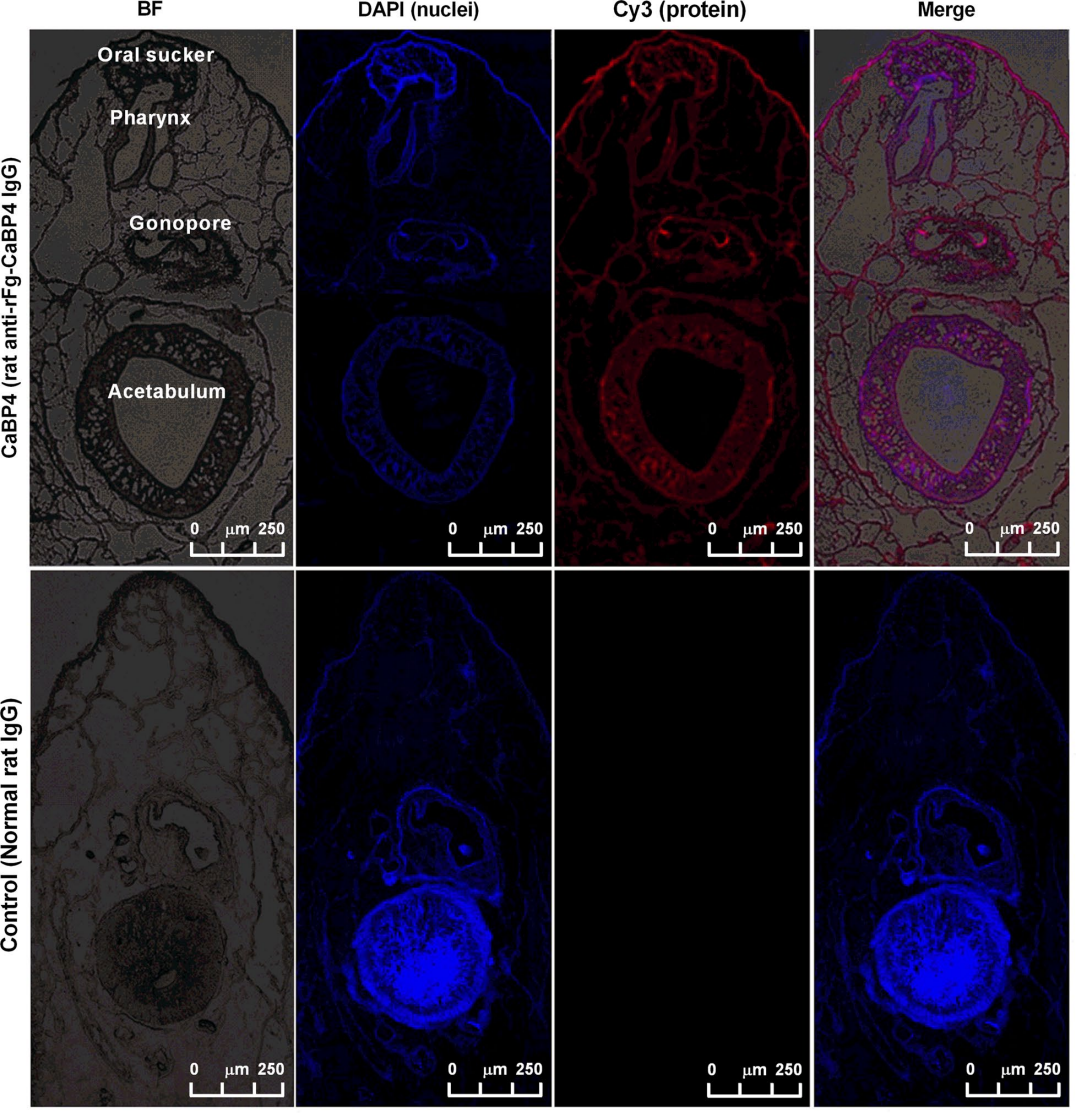
Immunolocalization of CaBP4 within adult *F. gigantica* flukes. The CaBP4 protein was detected in the tegument and inner lining of the oral sucker and ventral sucker (*Acetabulum*).* Red* denotes the target protein stained with cyanine dye 3 (*Cy3*)-conjugated secondary antibody.* Blue* denotes nuclei stained with 2-(4-amidinophenyl)-6-indolecarbamidine dihydrochloride (*DAPI*). No red fluorescence was detected in the control panel. *Merge* Combined DAPI and Cy3, *BF* bright fluorescence.* Scale bars* 250 μm

The bacterial cultures containing recombinant CaBP4 clones were induced with 1 mM IPTG at different time intervals and the transfected bacteria were collected by centrifugation. The sonicated bacterial lysate was purified by chromatography on Ni^2^^+^-nitrilotriacetic acid and the purified protein was resolved using 12% SDS-PAGE and produced a single band of about 26.2 kDa of Fg-CaBP4 protein (Fig. 3a). The high molecular mass of the purified protein, which differed from the size calculated based on deduced amino acids, was due to an additional 4-kDa fused protein of the expression vector. The immunogenicity of the Fg-CaBP4 protein was detected by western blotting using polyclonal antibodies generated against rFg-CaBP4 protein in Sprague–Dawley rats. The results showed an immunogenic reaction in samples probed with sera from immunized rats (rat anti-Fg-CaBP4), but no reaction was observed with the sera collected from non-immunized or normal rats (Fig. 3b). To examine the localization of native CaBP4 protein within adult *F. gigantica* flukes, anterior sections of the parasite including oral suckers, pharynx, genital pore and acetabulum were processed and stained for protein distribution. The results showed that antibodies raised against rFg-CaBP4 immunohistochemically detected the native protein CaBP4 in the oral suckers, pharynx, genital pore and acetabulum as well as the tegument of adult *F. gigantica* flukes, suggesting that CaBP4 is not only a tegumental protein, but also an excretory/secretory protein released from the host-parasite interface. As anticipated, no labeling was detected in control sections probed with antibodies derived from normal rats (Fig. 4).

### rFg-CaBP4 protein adhesion to the surface of goat monocytes

**Fig. 5:**
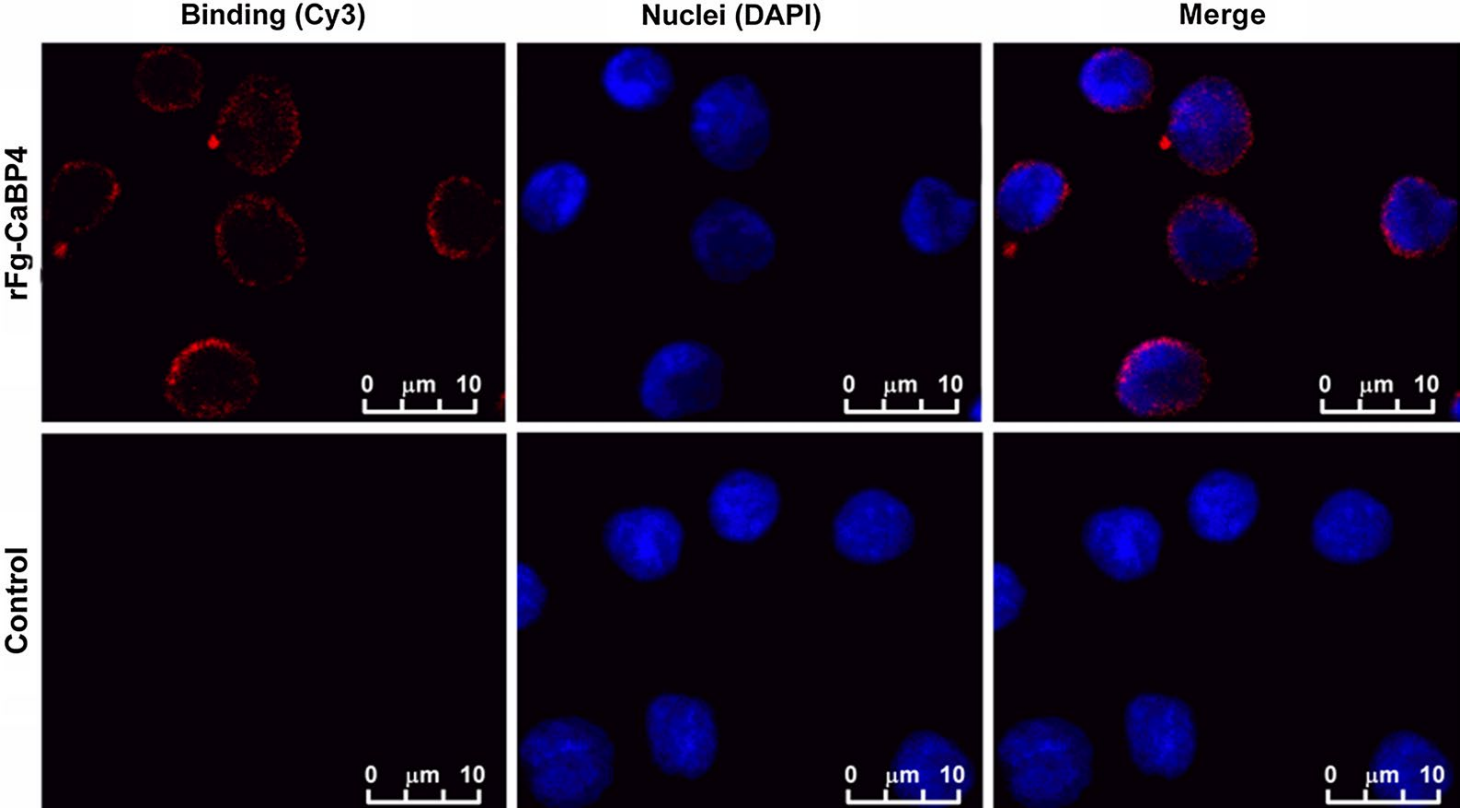
Binding of rFg-CaBP4 to the surface of goat monocytes. Goat monocytes were treated with rFg-CaBP4 (20 μg/ml) or left untreated for 60 min at 37 °C. All cells were fixed and incubated with rat anti-rFg-CaBP4 antibody followed by Cy3-labeled goat anti-rat IgG (*red*). The cell nuclei were counter labeled with DAPI (*blue*). The binding of rFg-CaBP4 to the surface of goat monocytes was observed as red staining with a confocal laser scanning microscopy.* Scale bars* 10 μm. For abbreviations, see Figs. 3 and  4

We evaluated the propensity of Fg-CaBP4 to attach to the surface of goat monocytes using an immunofluorescence assay. The results indicated that the Fg-CaBP4 protein could interact with monocytes by direct adherence to the cell surface, as indicated by the dense immunoreactivity and red fluorescence at the margin of monocytes. As expected, no fluorescence was detected in the control (untreated) monocytes (Fig. 5).

### Cytokine production in response to rFg-CaBP4

**Fig. 6:**
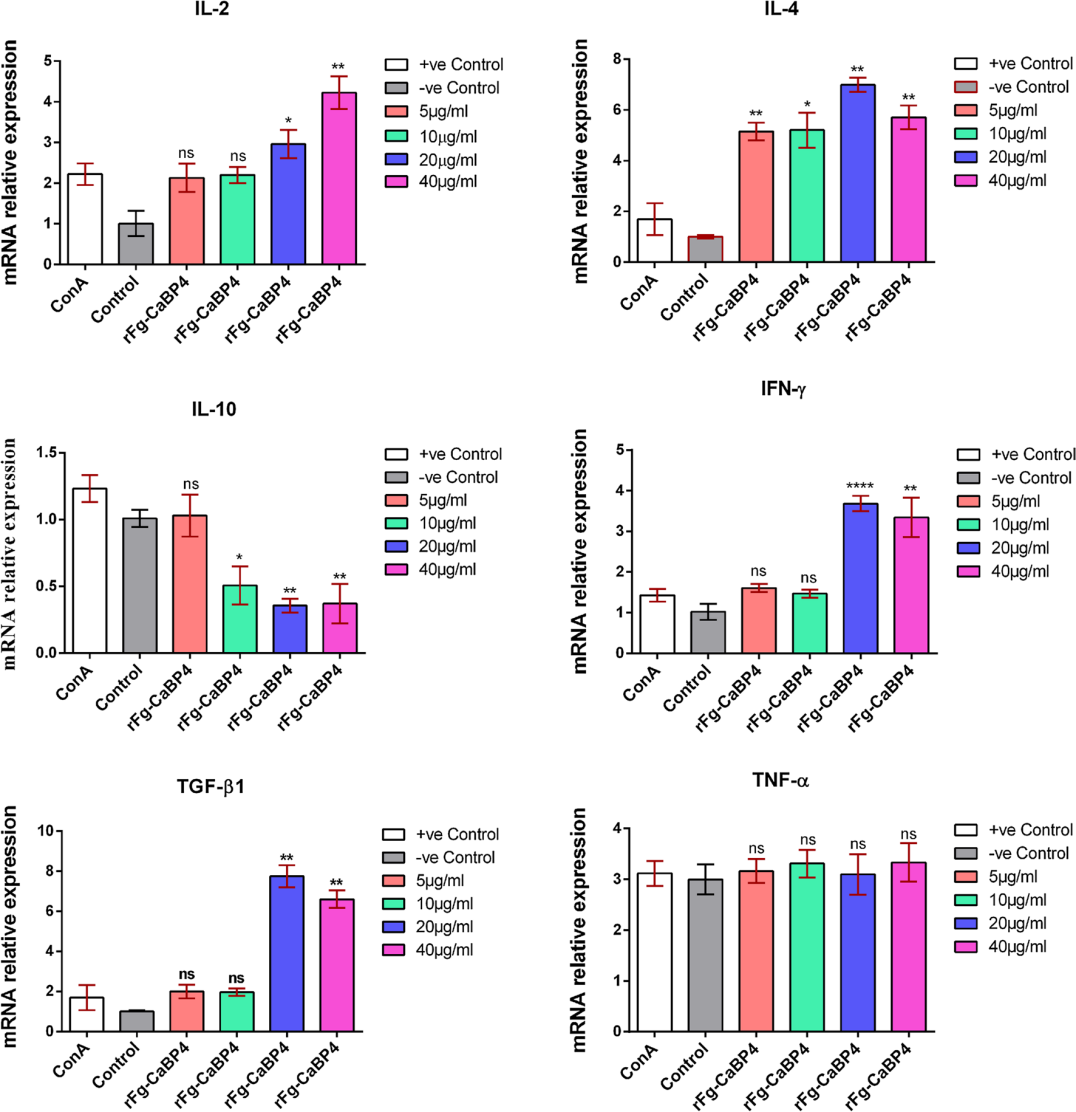
Effect of rFg-CaBP4 on goat monocyte cytokine production. Cells were stimulated with concanavalin A (*ConA*) (10 µg/ml) and incubated with rFg-CaBP4 for 26 h. Then, expression of the mRNAs of interleukin (*IL*)-2, IL-4, IL-10, IFN-γ, transforming growth factor-β1 (*TGF-β1*), and tumor necrosis factor-α (*TNF-α*) was quantified by reverse transcription-polymerase chain reaction. The data are presented as the mean ± SD from three independent experiments. *P < 0.05, **P < 0.01, ****P < 0.0001, ns [compared with the untreated (*Control*) group]

The monocytes induced with ConA, and incubated with different rFg-CaBP4 concentrations, showed increased levels of cytokines IL-2 [ANOVA, *F*_(5,12)_ = 33.53, *P* < 0.0001], IL-4 [ANOVA, *F*_(5,12)_ = 26.16, *P* < 0.0001], IFN-γ [ANOVA, *F*_(5,12)_ = 64.13, *P* < 0.0001] and TGF-β1 [ANOVA, *F*_(5,12)_ = 48.02, *P* < 0.0001]. However, the level of the IL-10 cytokine gradually decreased as the concentration of the rFg-CaBP4 protein increased [ANOVA, *F*_(5,12)_ = 10.62, *P* = 0.0004]. The secretion of the TNF-α cytokine was not significantly altered [ANOVA, *F*_(5,12)_ = 0.512, *P* = 0.762] compared to that of the control group (Fig. 6).

### rFg-CaBP4 increased the expression of MHC-II and decreased the expression of MHC-I

**Fig. 7:**
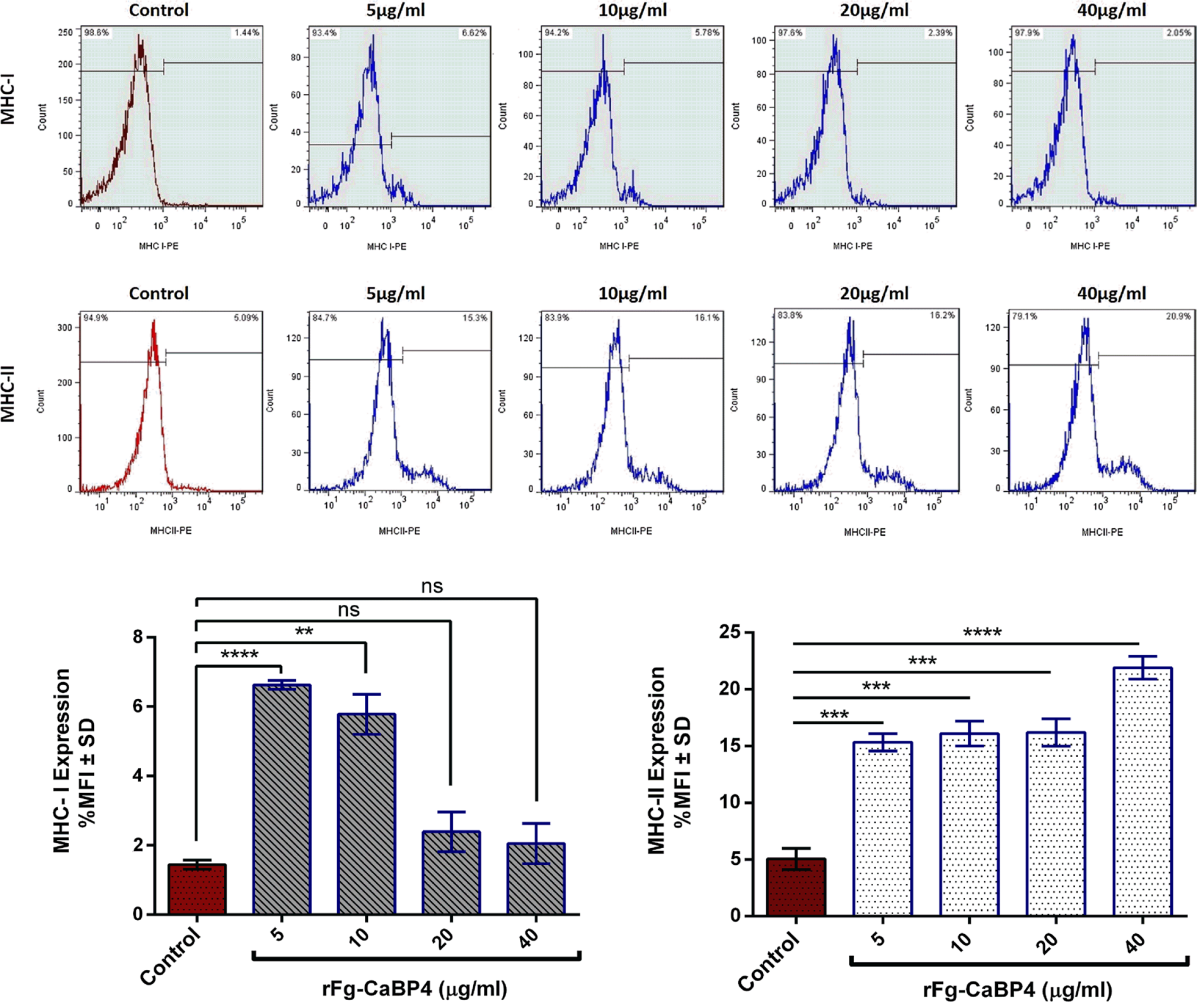
rFg-CaBP4-mediated differential expression of major histocompatibility complex (*MHC*) on the surface of goat monocytes. Monocytes were cultured in the presence of the indicated concentrations of rFg-CaBP4 and control buffer for 24 h. MHC class-I (MHC-I) and MHC-II expressions were measured by flow cytometric analysis and calculated as the percentage of mean fluorescence intensity (*MFI*). The data are presented as the MFI ± SD from three independent experiments. **P < 0.01, ***P < 0.001, ****P < 0.0001, ns (compared to untreated control)

The expression of MHC-I and MHC-II surface markers on goat monocytes was evaluated by flow cytometry, and the results showed that MHC-II^+^ expression on monocytes gradually increased in proportion to the concentrations of rFg-CaBP4 protein as follows: 5 μg/ml (*t*_*4*_ = 14.72, *P* = 0.0001), 10 μg/ml (*t*_*4*_ = 13.19, *P* = 0.0002), 20 μg/ml (*t*_*4*_ = 12.64, *P* = 0.0002), 40 μg/ml (*t*_*4*_ = 21.20, *P* < 0.0001) compared with the control group (Fig. 7). In contrast, the expression of MHC-I^+^ was higher in response to 5 μg/ml (*t*_*4*_ = 28.22, *P* < 0.0001) and 10 μg/ml (*t*_*4*_ = 7.342, *P* = 0.0018) concentrations, and reduced with increasing concentrations of rFg-CaBP4 at 20 μg/ml (*t*_*4*_ = 1.622, *P* = 0.1800) and 40 μg/ml (*t*_*4*_ = 1.032, *P* = 0.360), without any significant difference when compared to the control group (Fig. 7).

## Discussion

Fascioliasis impacts the livestock industry worldwide and imposes a public health threat. In order to accelerate the development of sustainable strategies to control fascioliasis, a better understanding of the modulatory effects of *Fasciola* spp. on host immune responses is needed. The ability of the liver flukes of the genus *Fasciola* to produce many excretory-secretory proteins including calcium-binding EF-hand proteins is one of the most critical strategies used by these parasites to manipulate the host immune responses and establish persistent infections. In this study, we successfully cloned and expressed the recombinant protein CaBP4. We isolated a complete ORF of CaBP4 from *F. gigantica* cDNA, which encoded a recombinant protein of ~ 26.2 kDa, including the 4-kDa fused peptides of the expression vector protein. The sequence similarity of the obtained protein was 96.86% to Fg-CaBP4, but lower, i.e. 48.39%, 48.37% and 47.85%, when compared with* F. hepatica* CaBP1, Fg-CaBP3 and tegumental antigen from schistosomes, respectively. Previous studies have shown that both native and rFg-CaBPs are antigenic molecules that induce a predominantly IgG1 response with lower IgE, IgG2a and IgG2b responses [9]. A previous study showed that *Opisthorchis viverrini* CaBP, which has a high structural resemblance to Fg-CaBP4. was distributed in the intestinal epithelium rather than tegumental region of the adult fluke [13]. In the case of *S. mansoni,* Sm-CaBP4 was located in the excretory system and intestinal lining [36]. In the present study, anti-rFg-CaBP4 antisera reacted with the native protein (CaBP4) in the fluke’s tegument and intestinal lining, reproductive and secretory/excretory structures, indicating that CaBP4, in addition to being a tegumental protein, is also an excretory/secretory protein released during infection. The excretory/secretory antigens interact with host immune cells through effector proteins, which are a prerequisite for immunoregulatory activity [37]. The proteomic profile of parasites contains various proteins with regulatory activities, which can challenge the host immune system by modulation, or suppression, of immune responses via the formation of receptor-ligand complexes on the surface of the host cell [38]. In our study, an immunofluorescence assay successfully demonstrated that rFg-CaBP4 protein can interact with and adhere to the surface of goat monocytes, providing further evidence that Fg-CaBP4 may play a role in immune regulation during the parasite-host relationship.

During the parasite-host relationship, Th1 and Th2 immune and inflammatory responses are associated with many cytokines and chemokines produced by many immune cell types (lymphocytes and monocytes), which perform different immunoregulatory functions [39]. In the present study, mRNA transcription levels of IL-2, IL-4, IL-10, IFN-γ, TGF-β1 and TNF-α were evaluated in host monocytes treated with different rFg-CaBP4 concentrations. To develop a tenacious infection in their mammalian definitive host, *Fasciola* species required a complex regulatory mechanism to challenge the host’s immune response, with Fg excretory-secretory proteins promoting various cellular and immunological tasks upon interaction with host immune cells [28]. *Fasciola* infection can induce a mixed Th1/Th2 immune response [40, 41]. Cell-mediated Th1 type immune and inflammatory responses are associated with IL-2, IFN-γ and TNF-α, along with monocyte activation, and have been detected in an intestinal nematode infection [42] and progression of the adaptive cellular immune response [43]. Recombinant proteins (rFg-CatB and rFg-Rab10) significantly promoted IL-2 and IFN-γ production in activated PBMCs [29, 44]. In another study, Th1 type cellular immune responses, characterized by the generation of IL-2 and IFN-γ mRNA transcripts, were observed in sheep challenged with *Haemonchus contortus* [45]. In this study, goat monocytes showed higher levels of IL-2 and IFN-γ cytokines in response to rFg-CaBP4 protein, suggesting that these cytokines may be linked to non-protective immune responses against fascioliasis. Th2 immune responses are characterized by IL-4 secretion, which acts as key mediator of humoral immunity to control helminth infection [46]. IL4 also participates in lymphocyte activation, differentiation, proliferation and antibody switching to IgG and IgE [47]. In this study, goat monocytes exposed to CaBP4 had elevated levels of IL-4, which promotes a Th2 type immune response during the parasite-host interaction. In a previous investigation, *F. hepatica* TegPs impaired host DCs’ maturation and functions by decreasing IL-10 and TNF-α secretions and cell surface markers [19, 20]. The immunosuppressive cytokine IL-10 was thought to have a negative influence on IL-2 and IFN-γ production [48], and exert an inhibitory impact on Th2 immune responses [49]. In this study, rFg-CaBP4 protein demonstrated a significant inhibition of the Th2 type immune response, as indicated by the reduction of the IL-10 cytokine level in host monocytes, and exerted a suppressive effect on the differentiation of regulatory T cells, which may be an immune modulatory role of Fg-CaBP4 against fascioliasis. TGF-β is implicated in various immune regulatory activities [50] and can inhibit T cell activation and differentiation [49]. Activated macrophages are key for immune responses with the production of the pro-inflammatory cytokine TNF-α in lipopolysaccharide-induced macrophages, which is required for the killing of pathogens [51]. In this study, binding of rFg-CaBP4 to goat monocytes increased the production of TGF-β1, which could be a mechanism employed by rFg-CaBP4 to facilitate immune evasion during host–parasite interactions. However, rFg-CaBP4 showed no significant effects on TNF-α secretion in goat monocytes. Our analysis on the production of Th1- and Th2-dependent cytokines in cultured monocytes showed that the rFg-CaBP4 protein can induce both humoral and cellular immune responses and is responsible for triggering Th1 and Th2 immune homeostasis during the parasite-host relationship.

MHC-I and -II molecules on the surface of antigen-presenting cells permit their exposure to extracellular antigens and are associated with parasite infection, antibody production and initiation of adaptive immune responses [52, 53]. In addition, cytokines—particularly IFN-γ and TGF-β—have been shown to have distinctive roles in MHC-I and -II gene expression on various host immune cells [54]. In the present study, we detected enhanced expression of MHC-II on monocytes treated with different concentrations of the rFg-CaBP4 protein. However, MHC-I expression was inhibited in response to a gradual increase in protein concentration. This indicated that the rFg-CaBP4 protein plays a vital role in the inhibition of parasitic infection by presenting antigen peptides to monocytes, and results in strong adaptive immune responses through increased levels of MHC-II molecule expression.

## Conclusions

We showed that rFg-CaBP4 protein is predominant in adult *F. gigantica* flukes and is localized in the tegument and internal structures of the flukes. The rFg-CaBP4 protein can actively bind to the surface of goat monocytes and modulates immune-associated cellular responses in stimulated goat monocytes. Our data offer new insight into the role of Fg-CaBP4 during interaction with innate immune cells* in vitro*. Further characterization of the role of Fg-CaBP4 and its potential utility in therapeutic interventions is needed.

## Supplementary Information

**Additional file 1: Table S1.** Primer sequences used for transcriptional analysis of Fg-CaBP4 by real-time PCR. **Table S2.** Primer sequences used for transcriptional analysis of cytokines by real-time PCR.

**Additional file 2: Figure S1.** N-terminal signal peptide prediction. The amino acid sequences of Fg-CaBP4 (GenBank: JN604670.1) was used to predict N-terminal signal peptides by the SignalP 4.1 Server. **Figure S2.** Fg-CaBP4 (GenBank: JN604670.1) was used to identify N-glycosylation sites. The consecutive amino acids in the sequence of Asn-Xaa-Ser/Thr are shown in* blue*. Asn predicted to be N-glycosylated is shown in* red*. Proteins without signal peptides are unlikely to be exposed to the N-glycosylation machinery and thus may not be glycosylated, even though they contain potential motifs. **Figure S3.** Transmembrane domain prediction using TMHMM Server v.2.0. The amino acid sequences of Fg-CaBP4 (GenBank: JN604670.1) was analyzed to predict transmembrane domain structure. There were no transmembrane domains predicted in this protein structure. **Figure S4.** Prediction of B cell epitopes for Fg-CaBP4. Protein sequence (GenBank: JN604670.1) was used for B cell epitope prediction using Bepipred Linear Epitope Prediction 2.0, which revealed 7 peptides of B cell epitopes. **Figure S5.** Protein sequence of Fg-CaBP4 (GenBank: JN604670.1) was used for prediction of T cell motifs. This analysis identified 12 potential epitopes in the target sequence.

## Data Availability

The datasets supporting the findings of this article are included within the article and its additional files.

## Abbreviations

CaBP: Calcium-binding protein
CaBP4: Tegumental calcium-binding EF-hand protein 4
cDNA: Complementary DNA
ConA: Concanavalin A
Cy3: Cyanine dye 3
DAPI: 2-(4-Amidinophenyl)-6-indolecarbamidine dihydrochloride
DCs: Dendritic cells
dpi: Days post-infection
FBS: Fetal bovine serum
IFN-γ: Interferon-γ
Ig: Immunoglobulin
IL: Interleukin
IPTG: Isopropyl-ß-D-thiogalactopyranoside
MHC: Major histocompatibility complex
mRNA: Messenger RNA
NCBI: National Center for Biotechnology Information
ORF: Open reading frame
PBMCs: Peripheral blood mononuclear cells
PBS: Phosphate buffered saline
Phyre2: Protein Homolog/analogY Recognition Engine v2.0
RPMI 1640: Roswell Park Memorial Institute 1640 culture media
RT-PCR: Reverse transcription-polymerase chain reaction
SDS-PAGE: Sodium dodecyl sulfate–polyacrylamide gel electrophoresis
TegPs: Tegumental proteins
TGF: Transforming growth factor
Th: Helper T cell
TNF-α: Tumor necrosis factor-α

## References

[1] Spithill TW, Carmona C, Piedrafita D, Smooker PM (2012). Prospects for immunoprophylaxis against *Fasciola hepatica* (liver fluke). Parasitic Helminths Targets Screens Drugs Vaccines.

[2] Piedrafita D, Spithill T, Smith R, Raadsma H (2010). Improving animal and human health through understanding liver fluke immunology. Parasite Immunol.

[3] Soliman MF (2008). Epidemiological review of human and animal fascioliasis in Egypt. J Infect Dev Countries.

[4] Young ND, Jex AR, Cantacessi C, Hall RS, Campbell BE, Spithill TW (2011). A portrait of the transcriptome of the neglected trematode, *Fasciola gigantica*—biological and biotechnological implications. PLoS Neg Trop Dis.

[5] Meemon K, Sobhon P (2015). Juvenile-specific cathepsin proteases in *Fasciola* spp.: their characteristics and vaccine efficacies. Parasitol Res.

[6] Brennan G, Fairweather I, Trudgett A, Hoey E, McConville M, Meaney M (2007). Understanding triclabendazole resistance. Exp Mol Pathol.

[7] Cheung S, Thomas CM, Timson DJ (2016). FhCaBP1 (FH22): a *Fasciola hepatica* calcium-binding protein with EF-hand and dynein light chain domains. Exp Parasitol.

[8] de Eguino ADR, Machín A, Casais R, Castro AM, Boga JA, Martín-Alonso JM, Parra F (1999). Cloning and expression in *Escherichia coli* of a *Fasciola hepatica* gene encoding a calcium-binding protein. Mol Biochem Parasitol.

[9] Subpipattana P, Grams R, Vichasri-Grams S (2012). Analysis of a calcium-binding EF-hand protein family in *Fasciola gigantica*. Exp Parasitol.

[10] Fitzsimmons CM, Jones FM, Stearn A, Chalmers IW, Hoffmann KF, Wawrzyniak J, Wilson S, Kabatereine NB, Dunne DW (2012). The *Schistosoma mansoni* tegumental-allergen-like (TAL) protein family: influence of developmental expression on human IgE responses. PLoS Negl Trop Dis.

[11] Kim YJ, Yoo WG, Lee MR, Kim DW, Lee WJ, Kang JM, Na BK, Ju JW (2012). Identification and characterization of a novel 21.6-kDa tegumental protein from *Clonorchis sinensis*. Parasitol Res.

[12] Zheng Y, Guo X, Su M, Chen X, Jin X, Ding J (2018). Identification of emu-TegP11, an EF-hand domain-containing tegumental protein of *Echinococcus multilocularis*. Vet Parasitol.

[13] Senawong G, Laha T, Loukas A, Brindley PJ, Sripa B (2012). Cloning, expression, and characterization of a novel *Opisthorchis viverrini* calcium-binding EF-hand protein. Parasitol Int.

[14] Thomas Charlotte M, Timson DJ (2016). A mysterious family of calcium-binding proteins from parasitic worms. Biochem Soc Trans.

[15] Zhang Z, Xu H, Gan W, Zeng S, Hu X (2012). *Schistosoma japonicum* calcium-binding tegumental protein SjTP22. 4. Immunization confers praziquantel schistosomulumicide and antifecundity effect in mice. Vaccine..

[16] Adams P, Aldridge A, Vukman K, Donnelly S, O'Neill S (2014). *Fasciola hepatica* tegumental antigens indirectly induce an M2 macrophage-like phenotype in vivo. Parasite Immunol.

[17] Jones MK, Gobert GN, Zhang L, Sunderland P, McManus DP (2004). The cytoskeleton and motor proteins of human schistosomes and their roles in surface maintenance and host–parasite interactions. BioEssays.

[18] Emmanoch P, Kosa N, Vichasri-Grams S, Tesana S, Grams R, Geadkaew-Krenc A (2018). Comparative characterization of four calcium-binding EF hand proteins from *Opisthorchis viverrini*. Korean J Parasitol.

[19] Hamilton CM, Dowling DJ, Loscher CE, Morphew RM, Brophy PM, O'Neill SM (2009). The *Fasciola hepatica* tegumental antigen suppresses dendritic cell maturation and function. Infect Immun.

[20] Haçarız O, Sayers G, Mulcahy G (2011). A preliminary study to understand the effect of *Fasciola hepatica* tegument on naïve macrophages and humoral responses in an ovine model. Vet Immunol Immunopathol.

[21] Islam MA, Große-Brinkhaus C, Pröll MJ, Uddin MJ, Aqter Rony S, Tesfaye D (2017). PBMC transcriptome profiles identifies potential candidate genes and functional networks controlling the innate and the adaptive immune response to PRRSV vaccine in Pietrain pig. PLoS ONE.

[22] Huang SY, Yue DM, Hou JL, Zhang XX, Zhang FK, Wang CR, Zhu XQ (2019). Proteomic analysis of *Fasciola gigantica* excretory and secretory products (FgESPs) interacting with buffalo serum of different infection periods by shotgun LC-MS/MS. Parasitol Res.

[23] Ehsan M, Wang W, Gadahi JA, Hasan MW, Lu M, Wang Y (2018). The serine/threonine-protein phosphatase 1 from *Haemonchus contortus* is actively involved in suppressive regulatory roles on immune functions of goat peripheral blood mononuclear cells. Front Immunol.

[24] Nicholson IC, Mavrangelos C, Fung K, Ayhan M, Levichkin I, Johnston A, Zola H, Hoogenraad NJ (2005). Characterisation of the protein composition of peripheral blood mononuclear cell microsomes by SDS-PAGE and mass spectrometry. J Immunol Methods.

[25] Wang Y, Lu M, Wang S, Ehsan M, Yan R, Song X, Xu L, Li X (2017). Characterization of a secreted macrophage migration inhibitory factor homologue of the parasitic nematode *Haemonchus contortus* acting at the parasite-host cell interface. Oncotarget.

[26] Zhang FK, Zhang XX, Elsheikha HM, He JJ, Sheng ZA, Zheng WB (2017). Transcriptomic responses of water buffalo liver to infection with the digenetic fluke *Fasciola gigantica*. Parasites Vectors.

[27] Hu RS, Zhang XX, Ma QN, Elsheikha HM, Ehsan M, Zhao Q, Fromm B, Zhu XQ (2021). Differential expression of microRNAs and tRNA fragments mediate the adaptation of the liver fluke *Fasciola gigantica* to its intermediate snail and definitive mammalian hosts. Int J Parasitol.

[28] Tian AL, Lu M, Calderón-Mantilla G, Petsalaki E, Dottorini T, Tian X (2018). A recombinant *Fasciola gigantica* 14-3-3 epsilon protein (rFg14-3-3e) modulates various functions of goat peripheral blood mononuclear cells. Parasites Vectors.

[29] Tian AL, Lu M, Zhang FK, Calderón-Mantilla G, Petsalaki E, Tian X (2018). The pervasive effects of recombinant *Fasciola gigantica* Ras-related protein Rab10 on the functions of goat peripheral blood mononuclear cells. Parasites Vectors.

[30] Tamura K, Stecher G, Peterson D, Filipski A, Kumar S (2013). MEGA6: molecular evolutionary genetics analysis version 6.0. Mol Biol Evol.

[31] Ehsan M, Gao W, Gadahi JA, Lu M, Liu X, Wang Y (2017). Arginine kinase from *Haemonchus contortus* decreased the proliferation and increased the apoptosis of goat PBMCs *in vitro*. Parasites Vectors.

[32] Bradford MM (1976). A rapid and sensitive method for the quantitation of microgram quantities of protein utilizing the principle of protein-dye binding. Anal Biochem.

[33] Lu M, Tian X, Tian A-L, Li C, Yan R, Xu L (2020). A novel α/β hydrolase domain protein derived from *Haemonchus contortus* acts at the parasite-host interface. Frontiers Immunol.

[34] Ehsan M, Gadahi JA, Lu M, Yan R, Xu L, Song X (2020). Recombinant elongation factor 1 alpha of *Haemonchus contortus* affects the functions of goat PBMCs. Parasite Immunol.

[35] Wang Y, Ehsan M, Huang J, Aimulajiang K, Yan R, Song X, Xu L, Li X (2020). Characterization of a rhodanese homologue from *Haemonchus contortus* and its immune-modulatory effects on goat immune cells in vitro. Parasites Vectors.

[36] Dunne DW, Webster M, Smith P, Langley JG, Richardson BA, Fulford AJ (1997). The isolation of a 22 kDa band after SDS-PAGE of *Schistosoma mansoni* adult worms and its use to demonstrate that IgE responses against the antigen(s) it contains are associated with human resistance to reinfection. Parasite Immunol.

[37] Gadahi JA, Wang S, Bo G, Ehsan M, Yan R, Song X, Xu L, Li X (2016). Proteomic analysis of the excretory and secretory proteins of *Haemonchus contortus* (HcESP) binding to goat PBMCs* in vivo* revealed stage-specific binding profiles. PLoS ONE.

[38] Ehsan M, Gadahi JA, Liu T, Lu M, Wang Y, Hasan MW (2020). Identification of a novel methyltransferase-type 12 protein from *Haemonchus contortus* and its effects on functions of goat PBMCs. Parasites Vectors.

[39] Grencis RK (2001). Cytokine regulation of resistance and susceptibility to intestinal nematode infection—from host to parasite. Vet Parasitol.

[40] Kumar N, Raina O, Nagar G, Prakash V, Jacob SS (2013). Th1 and Th2 cytokine gene expression in primary infection and vaccination against *Fasciola gigantica* in buffaloes by real-time PCR. Parasitol Res.

[41] Changklungmoa N, Phoinok N, Yencham C, Sobhon P, Kueakhai P (2016). Vaccine potential of recombinant cathepsinL1G against *Fasciola gigantica* in mice. Vet Parasitol.

[42] Muñoz-Carrillo JL, Contreras-Cordero JF, Muñoz-López JL, Maldonado-Tapia C, Muñoz-Escobedo JJ, Moreno-García MA (2017). Resiniferatoxin modulates the Th1 immune response and protects the host during intestinal nematode infection. Parasite Immunol..

[43] Cortés A, Muñoz-Antoli C, Esteban JG, Toledo R (2017). Th2 and Th1 responses: clear and hidden sides of immunity against intestinal helminths. Trends Parasitol.

[44] Chen D, Tian AL, Hou JL, Li JX, Tian XW, Yuan XD (2019). The multitasking *Fasciola gigantica* cathepsin B interferes with various functions of goat peripheral blood mononuclear cells *in vitro*. Front Immunol.

[45] Schallig HD (2000). Immunological responses of sheep to *Haemonchus contortus*. Parasitology.

[46] Lacroux C, Nguyen TH, Andreoletti O, Prevot F, Grisez C, Bergeaud JP (2006). *Haemonchus contortus* (Nematoda: Trichostrongylidae) infection in lambs elicits an unequivocal Th2 immune response. Vet Res.

[47] Nishio J, Honda K (2012). Immunoregulation by the gut microbiota. Cell Mol Life Sci.

[48] Moura VB, Lima SB, Matos-Silva H, Vinaud MC, Loyola PR, Lino RS (2016). Cellular immune response in intraventricular experimental neurocysticercosis. Parasitology.

[49] Taylor A, Verhagen J, Blaser K, Akdis M, Akdis CA (2006). Mechanisms of immune suppression by interleukin-10 and transforming growth factor-beta: the role of T regulatory cells. Immunology.

[50] Li MO, Wan YY, Sanjabi S, Robertson AKL, Flavell RA (2006). Transforming growth factor-β regulation of immune responses. Annu Rev Immunol.

[51] Xaus J, Comalada M, Valledor AF, Lloberas J, López-Soriano F, Argilés JM, Bogdan C, Celada A (2000). LPS induces apoptosis in macrophages mostly through the autocrine production of TNF-α. Blood J Am Soc Hematol.

[52] Keskinen P, Ronni T, Matikainen S, Lehtonen A, Julkunen I (1997). Regulation of HLA class I and II expression by interferons and influenza A virus in human peripheral blood mononuclear cells. Immunology.

[53] Chamuleau ME, Ossenkoppele GJ, Van De Loosdrecht AA (2006). MHC class II molecules in tumour immunology: prognostic marker and target for immune modulation. Immunobiology.

[54] Panek RB, Benveniste EN (1995). Class II MHC gene expression in microglia. Regulation by the cytokines IFN-gamma, TNF-alpha, and TGF-beta. J Immunol.

