# Supplementary material for: Fasciola gigantica tegumental calcium-binding EF-hand protein 4 exerts immunomodulatory effects on goat monocytes
Source: Parasit Vectors. 2021 May 22;14:276. doi: 10.1186/s13071-021-04784-5 (PMC8141160; doi:10.1186/s13071-021-04784-5)
Supplement: Supplementary file 1 — Additional file 1: Table S1. Primer sequences used for transcriptional analysis of Fg-CaBP4 by real-time PCR. Table S2. Primer sequences used for transcriptional analysis of cytokines by real-time PCR. [file 13071_2021_4784_MOESM1_ESM.docx]

**Additional file 1: Table S1.**

**Primer sequences used for transcriptional analysis of Fg-CaBP4 by real-time qPCR.**

| Primer name | Forward/reverse primer (5'→3') | Size (bp) | Amplification efficiency (%)* | Correlation coefficients (r^2^) |
| --- | --- | --- | --- | --- |
| β-Actin | F: CACCACACCTTCTACAAC  R: TCTGGGTCATCTTCTCAC | 106 | 95.41 | 0.9991 |
| Fg-CaBP4 | F: CGATGCTGACGAGAACGGA  R: CACGACCAGCTCTGACTTCC | 116 | 98.68 | 0.9993 |

* Amplification efficiency (%) = (10-1/slope -1) ×100

**Additional file 1: Table S2.**

**Primer sequences used for transcriptional analysis of cytokines by real-time qPCR.**

| Gene name | Forward/reverse primers (5′→ 3′) | Size (bp) | Amplification efficiency (%)* | Correlation coefficients (r^2^) |
| --- | --- | --- | --- | --- |
| GAPDH | F: CCTGGAGAAACCTGCCAAGT  R: GCCAAATTCATTGTCGTACCA | 214 | 99.02 | 0.9982 |
| IL-2 | F: CAAACGGTGCACCTACTTCA  R: AGCTTGAGGTTCTCGGGATT | 115 | 96.75 | 0.9985 |
| IL-4 | F: GTACCAGCCACTTCGTCCAT  R: GCTGCTGAGATTCCTGTCAA | 148 | 98.73 | 0.9994 |
| IL-10 | F: CCTTGTCGGAAATGATCCAG  R: AGGGCAGAAAACGATGACAG | 150 | 98.68 | 0.9993 |
| IFN-γ | F: GAACGGCAGCTCTGAGAAAC  R: GGTTAGATTTTGGCGACAGG | 131 | 98.02 | 0.9982 |
| TGF-β1 | F: CATGAACCGGCCCTTCCT  R: GAAGTCAATGTAGAGCTGACGAACA | 126 | 98.98 | 0.9996 |
| TNF-α | F: ATGAGCACTGAAAGCATGATCC  R: GAGGGCTGATTAGAGAGAGGTC | 217 | 97.85 | 0.9981 |

* Amplification efficiency (%) = (10-1/slope -1) ×100
