# Supplementary material for: Fasciola gigantica tegumental calcium-binding EF-hand protein 4 exerts immunomodulatory effects on goat monocytes
Source: Parasit Vectors. 2021 May 22;14:276. doi: 10.1186/s13071-021-04784-5 (PMC8141160; doi:10.1186/s13071-021-04784-5)

**Additional file 2: Figure S1.**

N-terminal signal peptide prediction. The amino acid sequences of Fg-CaBP4 (GenBank: JN604670.1) was used to predict N-terminal signal peptides by SignalP 4.1 Server.

**
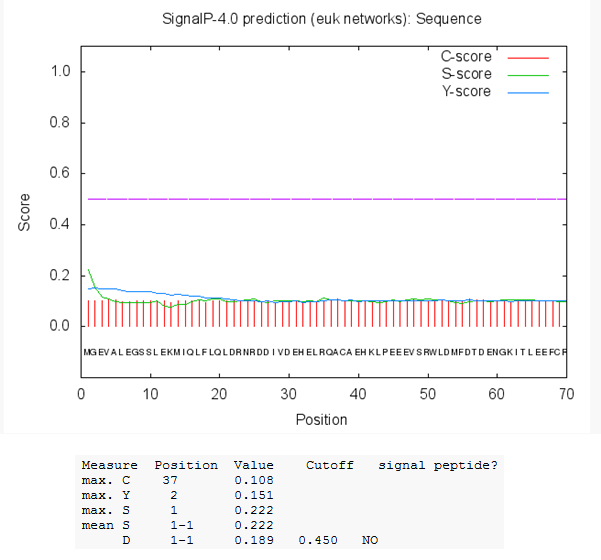
**

**Additional file 2: Figure S2.**

Fg-CaBP4 (GenBank: JN604670.1) was used to identify N-Glycosylation sites. The consecutive amino acids in the sequence for Asn-Xaa-Ser/Thr are highlighted in blue. Asparagines predicted to be N-glycosylated are highlighted in red. Proteins without signal peptides are unlikely to be exposed to the N-glycosylation machinery and thus may not be glycosylated, even though they contain potential motifs.

**
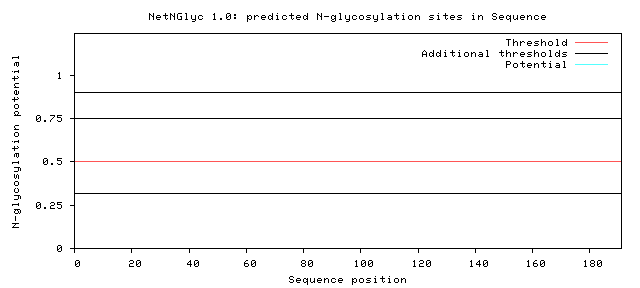
**

**Additional file 2: Figure S3.**

Transmembrane domains prediction using TMHMM Server v.2.0. The amino acid sequences of Fg-CaBP4 (GenBank: JN604670.1) was analysed to predict transmembrane domain in structure. There were no transmembrane domains predicted in this protein structure.


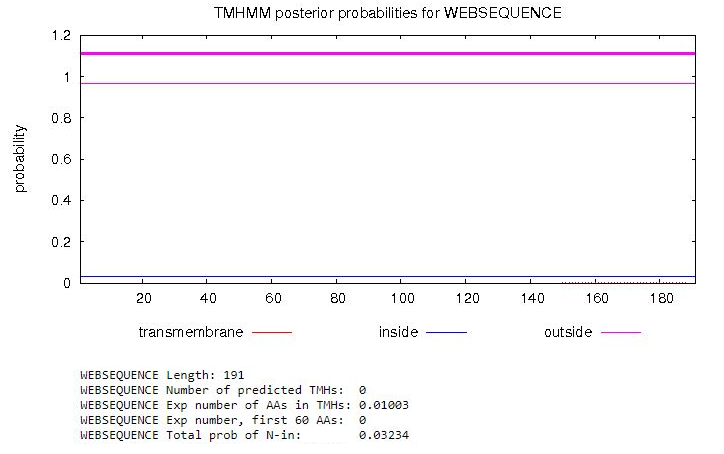


**Additional file 2: Figure S4.**

Prediction of B-cell epitopes for Fg-CaBP4. Protein sequence (GenBank: JN604670.1) was utilized for B-cell epitopes prediction using Bepipred Linear Epitope Prediction 2.0, that revealed 7 peptides of B cell epitopes.


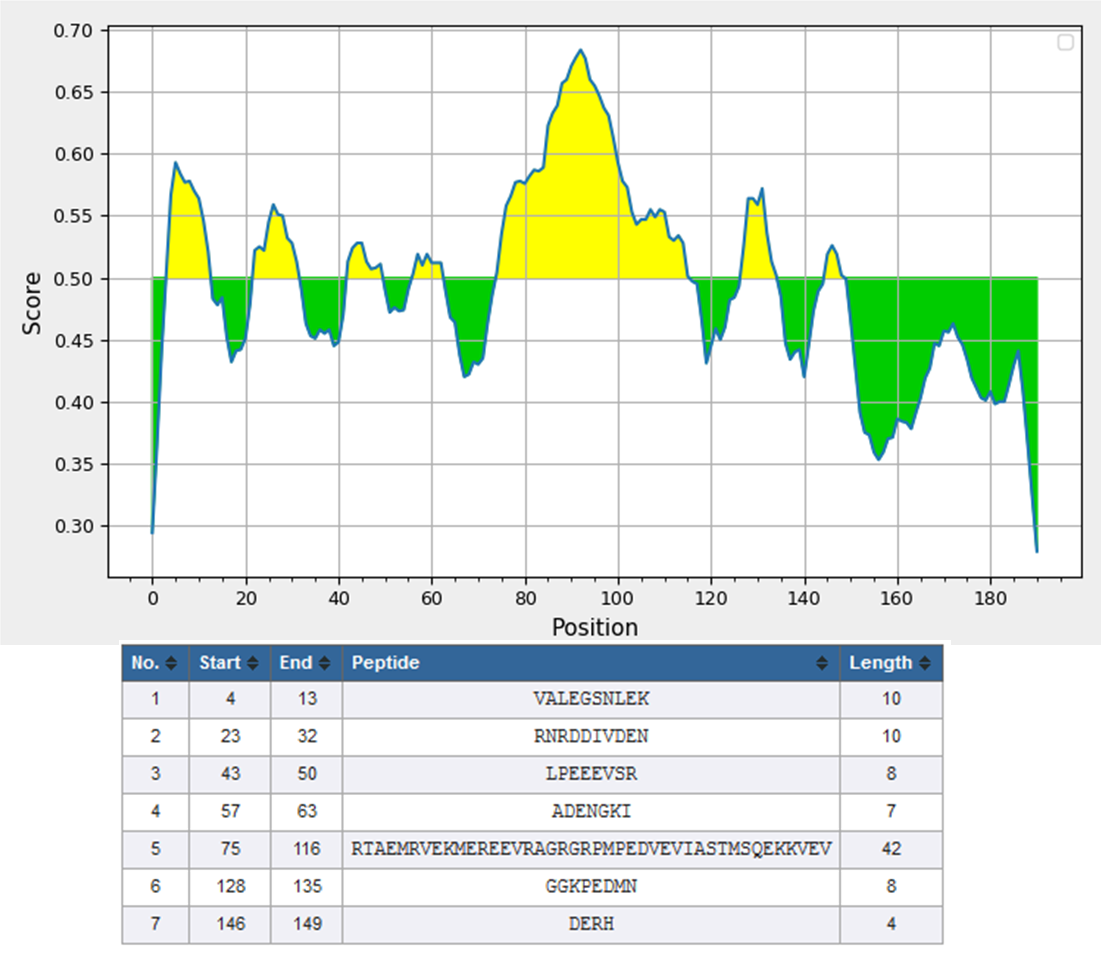


**Additional file 2: Figure S5.**

Protein sequence of Fg-CaBP4 (GenBank: JN604670.1) was utilized for prediction of T-cell motifs. The results showed that 12 potential T-Cell epitopes were found in the target sequence as shown below.


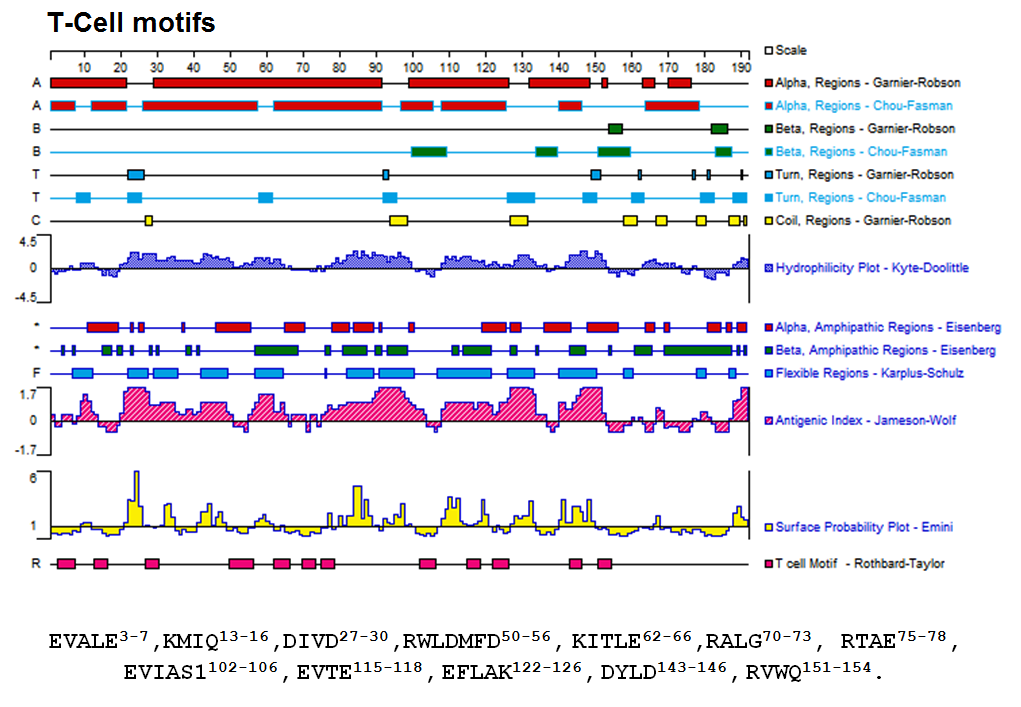

Supplement: Supplementary file 2 — Additional file 2: Figure S1. N-terminal signal peptide prediction. The amino acid sequences of Fg-CaBP4 (GenBank: JN604670.1) was used to predict N-terminal signal peptides by the SignalP 4.1 Server. Figure S2. Fg-CaBP4 (GenBank: JN604670.1) was used to identify N-glycosylation sites. The consecutive amino acids in the sequence of Asn-Xaa-Ser/Thr are shown in blue. Asn predicted to be N-glycosylated is shown in red. Proteins without signal peptides are unlikely to be exposed to the N-glycosylation machinery and thus may not be glycosylated, even though they contain potential motifs. Figure S3. Transmembrane domain prediction using TMHMM Server v.2.0. The amino acid sequences of Fg-CaBP4 (GenBank: JN604670.1) was analyzed to predict transmembrane domain structure. There were no transmembrane domains predicted in this protein structure. Figure S4. Prediction of B cell epitopes for Fg-CaBP4. Protein sequence (GenBank: JN604670.1) was used for B cell epitope prediction using Bepipred Linear Epitope Prediction 2.0, which revealed 7 peptides of B cell epitopes. Figure S5. Protein sequence of Fg-CaBP4 (GenBank: JN604670.1) was used for prediction of T cell motifs. This analysis identified 12 potential epitopes in the target sequence. [file 13071_2021_4784_MOESM2_ESM.docx]
